# Selection of hepatitis C virus resistant to ribavirin

**DOI:** 10.1186/1743-422X-8-402

**Published:** 2011-08-15

**Authors:** Dino A Feigelstock, Kathleen B Mihalik, Stephen M Feinstone

**Affiliations:** 1 Division of Viral Products, Center for Biologics Evaluation and Research, FDA, 29 Lincoln Drive, Bethesda, MD 20892, USA

## Abstract

**Background:**

Given the side effects associated with intravenous injections of interferon, an interferon-free regimen for the treatment of HCV infections is highly desirable. Recently published clinical studies show that interferon-free combination therapies containing ribavirin are efficacious, suggesting that an interferon-free therapy could be adopted in the near future. Therefore, understanding HCV resistance to ribavirin could be of major importance. In an approach to understand the effect of ribavirin on HCV replication and HCV resistance, we have selected a ribavirin resistant mutant of HCV *in vitro*.

**Methods:**

We serially passed the J6/JFH1 strain of HCV in Huh7D cells (a Huh7 cell derivative more permissive to HCV replication) in the presence of different concentrations of ribavirin. Virus replication was assessed by detection of HCV antigens by immunfluorscence of infected cells and titration of recovered virus present in the supernatant. cDNAs from virus RNA grown in 0 or 250 uM concentrations of ribavirin were synthesized by RT-PCR, and sequenced.

**Results:**

A concentration of 125 uM of ribavirin did not have a dramatic effect on HCV replication, while 500 uM of ribavirin lead to viral extinction. Concentrations of 250 uM of ribavirin dramatically reduced virus replication which was sustained over six passages. At passage seven viral resurgence began and over two passages the level of virus reached that of the wild type virus grown without ribavirin. Virus recovered from these cultures were more resistant to 250 uM ribavirin than wild type virus, and showed no difference in replication relative to wild type virus when grown in the absence of ribavirin. The ribavirin resistant virus accumulated multiple synonymous and non-synonymous mutations that are presently being analyzed for their relationship to ribavirin resistance.

**Conclusions:**

It is possible to select a ribavirin resistant mutant of HCV that can replicate to levels similar to wild type virus grown without ribavirin. Analysis of the mutations responsible for the ribavirin resistance may aid in understanding the mechanism of action of ribavirin.

## Background

While mono-therapy regimens of ribavirin have minimal effect on patients with chronic HCV infections, ribavirin clearly has a synergistic effect when combined with interferon. Therefore, understanding how ribavirin suppresses HCV replication and how HCV could escape the effect of ribavirin could be of major importance. Proposed mechanisms of action of ribavirin against HCV include a direct effect against the HCV RNA dependent RNA polymerase (NS5b); induction of misincorporation of nucleotides leading to lethal mutagenesis; depletion of intracellular guanosine triphosphate pools; alteration in the cytokine balance from a Th2 profile to a Th1 profile; and up-regulation of genes involved in interferon signaling [1,2]. Clinical studies showed that the efficacy of a interferon-free combination therapy involving a protease and a polymerase inhibitor can be strongly enhanced by adding ribavirin, suggesting a direct anti-viral action of ribavirin *in vivo *[3,4]. However, the mechanism of action of ribavirin is not completely understood. Availability of an HCV ribavirin resistant mutant could be useful to study the anti-HCV mechanism of action of ribavirin and HCV resistance. In addition, a ribavirin resistant HCV mutant could be used to study the cross-resistance of HCV to ribavirin and other nucleoside analogs. To date, the reported HCV ribavirin resistant mutants were based on HCV replicons and transient assays [5-7]. Here we report the selection of a ribavirin resistant HCV mutant that can replicate *in vitro *to high levels in the presence of high concentrations of ribavirin. The ribavirin resistant virus accumulated multiple mutations distributed throughout the HCV genome that are presently being analyzed.

## Results

### Selection of HCV resistant to ribavirin

**Figure 1:**
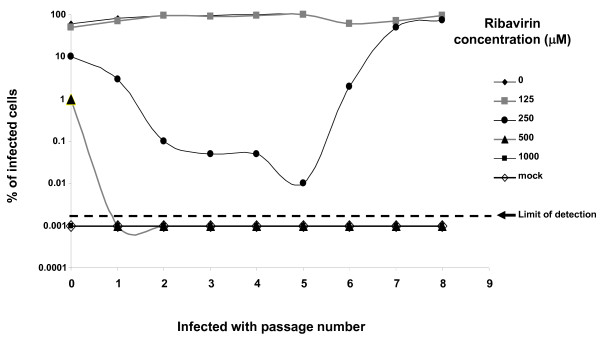
**Selection of HCV resistant to ribavirin**. J6/JFH1 virus was serially passaged in Huh7D cells in medium containing the indicated amount of ribavirin; at each passage intra-cellular HCV antigen was detected by immunfluorscence as described in the text. The percentage of infected cells was calculated by dividing the estimated amount of positive cells over the total number of cells per well.

In an approach to select a robust HCV resistant mutant we passed the J6/JFH1 strain of HCV[8] in the presence of different concentrations of ribavirin. Preliminary experiments showed that Huh7D cells could grow to confluence in concentrations of ribavirin up to 500 μM (although at a slower rate than in absence of ribavirin) and concentrations of 250 μM ribavirin and higher affected the growth of HCV in persistently infected cells (not shown). In addition, it has been reported that Huh7 cells can acquire resistance to ribavirin by restricting ribavirin uptake[6,9]. Therefore, in order to avoid the selection of ribavirin-resistant-cells that would allow the virus to grow in ribavirin containing medium (without being an authentic ribavirin resistant virus), we subjected the J6/JFH1 virus to successive seven days interval passages in naïve Huh7D cells (a highly permissive clone derived from Huh7 cells[10]) that had not previously been exposed to ribavirin as described in the material and methods section. As shown in Figure 1, a concentration of 125 μM ribavirin did not have a detectable effect on HCV replication, while 500 μM and 1000 μM ribavirin lead to viral extinction. Concentrations of 250 μM of ribavirin dramatically reduced virus replication which was sustained over six passages. At passage seven, viral resurgence began and over the next two passages the level of virus reached that of the virus grown without ribavirin. The high percentage of infected cells observed at passage eight in cultures treated with 250 μM ribavirin suggested that the recovered virus was resistant to ribavirin. The virus recovered from passage seven in 250 μM ribavirin was grown in naïve Huh7D cells (not treated with ribavirin) for a week and the supernatant from this culture was harvested, titrated to 6.5 × 10^4 ^ffu/ml, and designated HCV-RR1.

Of note, in some experiments J6/JFH1 resurged after several passages in 250 μM ribavirin (as in Figure 1), while in others it resurged faster or it did not resurge at all (not shown). The level of infected cells before virus resurgence could explain the observed differences: in some instances, it is possible that no infectious virus survived after a few passages in ribavirin and therefore the opportunity to select a resistant mutant was lost. In addition, we observed variability in viral titers which we don't fully understand.

### Virus recovered from ribavirin treated cells is resistant to ribavirin

**Figure 2:**
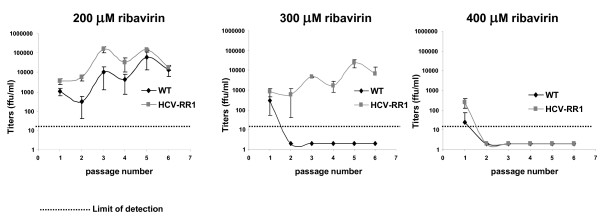
**HCV recovered from ribavirin treated cells is resistant to ribavirin**. J6/JFH1 and HCV-RR1 were serially passaged in Huh7D cells in medium containing the indicated concentration of ribavirin; at each passage HCV titers in the supernatants were obtained as described in the text. Titers are expressed as the mean number of foci of each of four replicates. Error bars represent the standard deviation.

In order to test if HCV-RR1 was a truly ribavirin resistant virus, J6/JFH1 and HCV-RR1 were grown in 200, 300, or 400 μM ribavirin by serially passaging the viruses every 5 to 7 days in naïve Huh7D cells as described for the experiment shown in Figure 1. Virus growth was assessed by IF (not shown) and by titration of the supernatants from each passage, as previously described[10](Figure 2). HCV-RR1 grew slightly better than J6/JFH1 in 200 μM ribavirin. In 300 μM ribavirin, HCV-RR1 grew reaching titers of 10^4 ^ffu/ml, while J6/JFH1 did not survive. Neither virus survived the 400 μM ribavirin treatment. This result indicates that viruses recovered from the 250 μM ribavirin treated cells (Figure 1) were resistant to ribavirin. We further subjected HCV-RR1 to ten passages in Huh7D cells treated with 250 μM ribavirin. The supernatant recovered from P10 cultures was harvested, titrated to 4.3 × 10^3 ^ffu/ml, and designated HCV-RR2.

### Kinetics of virus growth in 300 μM ribavirin for one week

**Figure 3:**
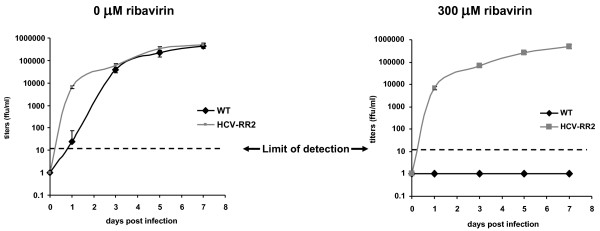
**Growth of HCV in 300 μM ribavirin for one week**. Huh7D cells were mock infected or infected with wild type J6/JFH1 or HCV-RR2 at a m.o.i. of 0.01. After 6 hours, cells were washed with growth medium three times and passed by tripsinization to 12 well plates containing a final concentration of zero or 300 μM ribavirin. Cells were collected at the indicated time points and frozen at -70°C. Virus was tittered as described in the text. Titers are expressed as the mean number of foci of each of four replicates. Error bars represent the standard deviation.

We confirmed the ribavirin resistant phenotype of HCV-RR1 by determining the kinetics of the growth of J6/JFH1 and HCV-RR2 in a concentration of zero or 300 μM ribavirin for one week as described in the material and methods section. J6/JFH1 and HCV-RR2 grew to similar levels in medium containing no ribavirin (HCV-RR2 showed a faster growth kinetics in this experiment). In medium containing 300 μM of ribavirin, J6/JFH1 did not grow, while HCV-RR2 attained titers similar to HCV-RR2 grown without ribavirin (Figure 3).

### Virus resistant to ribavirin acquired synonymous and non-synonymous mutations

In order to find out if ribavirin resistant viruses acquired mutations, viral RNA from HCV-RR1 and HCV-RR2 was extracted, reverse transcribed, amplified by PCR, and sequenced. In the coding region, HCV-RR1 acquired 26 nucleotide substitutions (relative to wild type J6/JFH1), 11 of which predicted amino-acid changes. These mutations were located in the core, E2, NS2, NS5a, and NS5b proteins. The HCV-RR2 sequence showed the same 26 substitutions found in HCV-RR1 and 12 additional nucleotide substitutions, 3 of which resulted in amino-acid changes (one additional substitution located in NS3 and two in NS5b) (see Table 1). We note that we found in both viruses mutation T2667C leading to amino-acid substitution Phe to Ser in the P7 protein (F716S in the HCV polyprotein). However, we also found this mutation in J6/JFH1 viruses that were passed without ribavirin (unpublished results), suggesting that F716S is not a ribavirin resistant determinant. The sequence of the 5' UTR from HCV-RR1 and HCV-RR2 (starting at nucleotide 29) showed no mutations. Sequence of the 3' UTR from HCV-RR1 showed no mutations through nucleotide 9481, while HCV-RR2 showed two mutations at the start of the polypyrimidine [poly (U/UC)] tract: C9480T and C9481T. Although we were able to detect the presence of the poly (U/UC) tract (nucleotides 9478 through 9558) in both viruses, we couldn't accurately determine the exact number of U/UCs. In addition, we couldn't accurately resolve the end of the poly (U/UC) tract, from nucleotide 9540 till nucleotide 9558. No mutations were observed from nucleotide 9558 till nucleotide 9640.

**Table 1:** Mutations observed in HCV resistant to ribavirin

Nucleotide position*	Nucleotide in J6/JFH1	Nucleotide in HCV-RR1	Nucleotide in HCV-RR2	Protein	Amino-acid position*^&^	Amino-acid in J6/JFH1	Amino-acid in HCV-RR1	Amino-acid in HCV-RR2
367	A	C	C	core	9	R	S	S
376	A	G	G					
572	A	G	G		78	K	E	E
581	T	C	C		81	Y	H	H
773	G	A	A		145	G	S	S
901	C	T	T					
								
1021^#^	C	T	T	E1				
								
1691	G	A	A	E2	451	G	R	R
1855	T	C	C					
1996	C	C	T					
2492	G	A	A		718	V	I	I
2667^^^	T	C	C	P7	776	F	S	S
								
2803	G	G	A	NS2				
3050	T	C	C		904	Y	H	H
3369	T	G	G		1010	L	R	R
								
3520	C	C	T	NS3				
3691	C	T	T					
4937	G	G	A		1533	D	D	N
5143	C	T	T					
								
5473	G	G	A	NS4a				
								
5686	G	G	A	NS4b				
5704	C	C	T					
5753	T	C	C					
								
6583	C	T	T	NS5a				
7216	C	T	T					
7320	C	T	T		2327	P	L	L
7441	G	A	A					
7573	T	C	C					
7652	A	G	G		2438	T	A	A
								
7710	G	A	A	NS5b	2457	S	N	N
7996	C	T	T					
8065	A	G	G					
8188	C	C	T					
8286	C	C	T		2649	A	A	V
8359	A	G	G					
8911	T	T	C					
8971	C	C	T					
9018	T	T	C		2893	V	V	A
9082	T	C	C					

* nomenclature is according to the JFH1 sequence, accession number AB047639.

**^&^**Only amino-acids where a substitution in HCV-RR viruses was found are indicated.

^# ^Position 1021 is a mix of C and T in HCV-RR1 and HCV-RR2.

^^ ^T2667C was also found in J6/JFH1 viruses that were passed without ribavirin (unpublished results).

## Discussion

In this study we obtained an HCV mutant that grows in cultured cells in the presence of medium concentrations of up to 300 μM ribavirin. We were able to derive this ribavirin resistant virus by successive weekly passages of the J6/JFH1 strain in Huh7D cells that were treated with medium containing 250 μM ribavirin (Figure 1). During the first six passages, the percentage of infected cells decreased till almost the extinction of the virus; after passage six, virus resurged and by passage nine more than 90% of the cells were infected. This initial result suggested that the recovered virus was resistant to ribavirin. We confirmed the ribavirin resistant phenotype of the recovered virus by infecting naïve cells and showing that it can grow to high titers even in a concentration of 300 μM ribavirin (Figures 2 and 3). The recovered virus acquired synonymous and non-synonymous mutations that were distributed all along the genome (Table 1). These mutations were maintained after nine passages in medium without ribavirin, with the exception of C/T1021T silent mutation in E1 (that is a mix of C and T in HCV-RR and reverted to C by passage nine) and C8286T that reverted to a mix of C and T (data not shown).

The NS5b from HCV can use ribavirin triphosphate as a nucleotide substrate, incorporating ribavirin monophosphate opposite to pyrimidines (U or C) but not opposite to purine (A or G) residues, leading to transition mutations[11,12]. Out of the 38 mutations we found in HCV-RR2, 36 were transitions and two were transversions (Table 1) possibly reflecting the mutagenic effect of ribavirin during the initial passages.

One or more of the observed mutations in HCV-RR viruses could be responsible for the ribavirin resistant phenotype. The above mentioned studies and studies showing that ribavirin increases the mutation rate of HCV in cultured cells [13-16] and *in vivo *[15,17] (although others did not observe an increase in mutation frequency[18,19]) point to NS5b as a possible genetic determinant of resistance to ribavirin. In our newly described HCV-RR viruses we found mutation Ser to Asn at amino-acid 15 of NS5b. According to crystallographic studies amino-acid 15 is located in the finger domain of NS5b[20-22]. Interestingly, this region is structurally related to the region where a single point mutation (G64S in the RNA polymerase) confers resistance to the catastrophic error[23] caused by ribavirin in poliovirus[24-26].

We do not know at this time the *in vivo *relevance of our results. It can be argued that in chronically infected patients subjected to ribavirin and interferon combination therapy, the plasma ribavirin concentration, estimated to be around 10 μM[27-29], is lower than the concentrations we needed to suppress HCV replication in our system. However, we don't know the concentration of ribavirin in hepatocytes, nor do we know the intracellular concentration of ribavirin in Huh7D cells. In one clinical report[30], while plasma ribavirin concentrations were of 8.8 μM, erythrocyte ribavirin concentrations were of 1389 μM, more than 150 times higher. This observation suggests that intracellular ribavirin could also be higher in cells other than erythrocytes, i.e. hepatocytes, compared to plasma.

## Conclusion

Ribavirin has proven to be effective for the treatment of HCV chronically infected patients when combined with interferon. Given the side effects associated with intravenous injections of interferon, an interferon-free regimen for the treatment of HCV infections is highly desirable. The study of HCV resistance to ribavirin could be specially important for an interferon-free era. In this report, we show that it is possible to select a ribavirin resistant mutant of HCV *in vitro *that can replicate to similar levels to virus grown without ribavirin. Analysis of the mutations responsible for the ribavirin resistance phenotype, currently underway, may aid in understanding the mechanism of action of ribavirin.

## Methods

### Cells

Huh7D cells, a highly permissive clone derived from Huh7 cells^8 ^were grown in DMEM (Gibco) supplemented with 10% bovine calf serum (Atlanta Biologicals), L-glutamine (Gibco), penicillin, and streptomycin (Gibco)..

### Viruses

To obtain the J6/JFH1, plasmid pFL- J6/JFH1[8] (a plasmid coding for full length J6/JFH1 virus, kindly provided by Dr Charles Rice) or the corresponding recombinant plasmids were cut with restriction enzyme Xba1 and transcribed with T7 RNA polymerase following the manufacturer instructions (T7 Megascript AMBION). *In vitro *transcribed HCV RNA was mixed with lipofectamine 2000 (Invitrogen) in Optimem (Gibco) and transfected into Huh7D cells. HCV replication was confirmed by immunfluorscence as described below. Virus from transfected cells was amplified by inoculating the supernatants from transfected cells into naïve Huh7D cells. Virus stocks were made from the supernatants of infected cells and titered as described below.

### Antibodies

Monoclonal antibody 6G7 directed to the HCV core protein was kindly provided by Henry H. Hsu and Harry B. Greenberg (Stanford University, Palo Alto Veterans Administration Medical Center, Palo Alto, CA)[31].

### Infection of Huh7D cells with wild type or ribavirin resistant J6/JFH1 viruses and treatment with ribavirin

Huh7D cells grown in 48-well plates were mock infected or infected with the indicated virus at a moi of 0.01. At 5 to 7 hours post infection, medium was replaced with 500 μl of medium containing the indicated amount of ribavirin. At 6 or 7 days post infection, 200 μl of the supernatants were used to inoculate naïve Huh7D cells that were seeded the day before, and at 5 to 7 hours post infection medium was replaced with 500 μl of medium containing the corresponding amount of ribavirin. The rest of the supernatants were stored at -70°C. This procedure was repeated for the indicated number of passages. HCV antigen was detected in the remaining monolayers by immunfluorscence (IF) and HCV titers were obtained from the supernatants as described below.

### Growth of HCV in 300 μM ribavirin for one week

Huh7D cells grown in 6-well plates were mock infected or infected with J6/JFH1 or HCV-RR2 at a moi of 0.01. At 6 hours post infection, cells were washed three times with medium and split into 12-well plates containing a final concentration of zero or 300 μM of ribavirin. At 0, 1, 3, 5, and 7 dpi the 12-well plates were frozen at -70°C. Total virus from each time point was recovered by freezing and thawing the cells 3 times and titered.

### Titration of viruses

To obtain virus titers, monolayers of Huh7D cells grown in 96 well plates were infected with 100 μl of 10-fold serial dilutions of the corresponding virus (in quadruplicates). At three days post infection, viral antigen was detected by IF as described below. Foci were counted and titers were expressed as the mean number of foci of each of the four replicates +/- the standard deviation.

### Detection of HCV antigen by immunfluorscence (IF)

Infected cells were fixed with methanol, washed with 1×PBS, blocked with a solution containing 1% BSA and 0.2% non-fat milk in 1×PBS, washed with 1×PBS, treated with a 1:500 dilution of monoclonal antibody 6G7 in 0.05% tween 20 in 1×PBS, washed with 1×PBS, stained with FITC-conjugated goat anti-mouse antibody (KPL), washed with 1×PBS, and observed in the microscope under UV light. The percentage of positive cells was determined by dividing the estimated amount of positive cells over the total number of cells per well.

### Sequencing of ribavirin resistant viruses

Viral RNA was extracted from virus stocks using Trizol reagent as recommended by the manufacturer (Invitrogen). cDNA was synthesized using SuperScript III reverse transcriptase and random primers (Invitrogen). PCR amplification of the HCV genome was performed using the Expand High Fidelity PCR system (Roche) as recommended by the manufacturer and the following sets of primers: 2a10+ and 2a1260-, 2a1101+ and 2a2690- (5'- CCTTGATGTACCAAGCAGCC-3'), 2a2431+ (5'-CCAAAACATCGTGGACGTAC-3') and 2a3980- (5'-AAGTGGGAGACCTTGTAACA-3'), 2a3721+ (5'-CAAGTGTGGAGCCGTCGACC-3') and 2a6320- (5'-CTGTCAAGATGGTGCAAACC, 2a6181+ (5'-TGTGACCCAACTACTTGGCT-3') and negNS5b15mut, 2a6781+ (5'-TGAGGTCTCGTTCTGCGTTG-3') and 2a8760- (5'-GATGTTATTAGCTCCAGGTC-3'), 2a8521+ (5'-AACCACTAGCATGGGTAACA-3') and 2a9480- (5'- GAACAGTTAGCTATGGAGTG-3'), 2a9001+ (5'- TGAGATGTATGGATCAGTAT -3') or 2a9040+ (5'- CTTCCAGCCATAATTGAGAG -3') and 2a9660- (5'-CCAGTTACGGCACTCTCTGC -3') or 2a9678- (5'-ACATGATCTGCAGAGAGACC-3'). PCR products were run in agarose gels and purified using gene-elute agarose gel columns (Sigma) or the QIAquick PCR purification Kit (Qiagen) and sequenced using the BigDye terminator v3.1 cycle-sequencing kit (Applied Biosystems) and the 3130xl Genetic analyzer (Applied Biosystems). In addition to the oligos used for PCR, we used the following oligos for sequencing: 2a4280- (5'-AGGCGCCGCTAGCGCAGCCC-3'), 2a4431+ (5'-CCGATATAGAAGAGGTAGGC-3'), 2a5051+ (5'-CACATAGACGCCCACTTCCT-3'), 2a7860- (5'-TCATAATGGGCGTCGAGCAC-3') and 2a9561+ (5'-CTTATTCTACTTTCTTTCTT-3').

## Competing interests

The authors declare that they have no competing interests.

## Authors' contributions

DAF conceived the study, its design, and coordination. DAF and KBM performed the experiments. DAF drafted the manuscript with the help of SMF and KBM. SMF supervised the study. All authors approved the final version.
